# Supplementary material for: Transmissive metasurface with 3.5-μm-thick liquid crystals for subterahertz-wave dynamic beamforming
Source: Commun Eng. 2026 Mar 9;5:56. doi: 10.1038/s44172-026-00635-2 (PMC13013568; doi:10.1038/s44172-026-00635-2)
Supplement: Supplementary file 2 — Supplementary notes [file 44172_2026_635_MOESM2_ESM.pdf]

Supplementary notes:

Transmissive metasurface with 3.5-μm-thick liquid crystals for subterahertz-wave dynamic beamforming

Daisuke Kitayama,[1,*] Hibiki Kagami,[1] Adam Pander,[1] Yuto Hama,[2] and Hiroyuki Takahashi[1]

[1]Device Technology Labs., NTT, Inc., 3-1 Morinosato-Wakamiya, Atsugi, Kanagawa 243-0198, Japan
[2]Department of Electrical and Computer Engineering, Yokohama National University, 79-5 Tokiwadai, Hodogaya, Yokohama, Kanagawa 240-8501, Japan
*daisuke.kitayama@ntt.com

CONTENTS

Note 1. Required size for ideally designed/controlled metasurface

Note 2. Quality factor of the antisymmetric mode

Note 3. Excited dipole in the circulating-current mode of the S-SRRs

Note 4. Effect of material properties on resonant characteristics

Note 5. Channel model for metasurface-assisted path

Note 6. Far-field validation with reduced aperture

Note 7. Polarization and incident angle dependency of the S-SRRs

Note 8. Line-by-line matrix control

Note 9. Estimated diffraction efficiency for RIS with all-on configuration

Note 10. Effect of unit cell size on the steering characteristics

Note 11. Near-field distribution in the magnetic-field plane

Note 12. Measurement of response time of LC orientation change

Note 13. Detailed geometrical parameters of unit cells

Note 14. LC material information

Note 15. Effect of alignment error on the S-SRRs' characteristics

Note 16. Measurement uncertainty and positioning sensitivity

1. Required size for ideally designed/controlled metasurface

In general, the propagation loss between a transmitter (Tx) and receiver (Rx) through a metasurface, or reconfigurable intelligent surface (RIS), differs from the free space propagation loss (FSPL) for the same total path lengt[1]. Here, we show the required size of the RIS to minimize the total propagation loss of the path through the square metasurface to a level commensurate with the FSPL along the total propagation path length.

Let d denote the distance between the transmitter and receiver. If there is a direct path, the corresponding FSPL is given by

$$P_{\mathrm{direct}} = \left(\frac{4\pi d}{\lambda}\right)^2, \qquad (S1)$$

where λ is the wavelength.

Next, we consider the link via RIS with aperture A. Let $d_{\text{Tx-RIS}}$ denote the distance between the Tx and RIS and $d_{\text{RIS-Rx}}$ the distance between the RIS and Rx. The array gain of the antenna with aperture A is expressed as

$$G = \frac{4\pi A}{\lambda^2}. \qquad (S2)$$

When the RIS with an array gain corresponding to its effective aperture area $A\cos\phi_{\mathrm{Tx-RIS}}$ receives arriving waves, the path loss between the Tx and RIS is given by

$$P_{\mathrm{Tx-RIS}} = \left(\frac{4\pi d_{\mathrm{Tx-RIS}}}{\lambda}\right)^2 \cdot \frac{1}{G} = \frac{4^2\pi^2 {d_{\mathrm{Tx-RIS}}}^2}{\lambda^2} \cdot \frac{\lambda^2}{4\pi A\cos\phi_{\mathrm{Tx-RIS}}} = \frac{4\pi {d_{\mathrm{Tx-RIS}}}^2}{A\cos\phi_{\mathrm{Tx-RIS}}}, \quad (S3)$$

where $\phi_{\mathrm{Tx-RIS}}$ represents the incident angle on the RIS plane.
Similarly, the path loss between RIS and Rx is given by

$$P_{\mathrm{RIS-Rx}} = \left(\frac{4\pi d_{\mathrm{RIS-Rx}}}{\lambda}\right)^2 \cdot \frac{1}{G} = \frac{4^2\pi^2 {d_{\mathrm{RIS-Rx}}}^2}{\lambda^2} \cdot \frac{\lambda^2}{4\pi A\cos\phi_{\mathrm{RIS-Rx}}} = \frac{4\pi {d_{\mathrm{RIS-Rx}}}^2}{A\cos\phi_{\mathrm{RIS-Rx}}}. \quad (S4)$$

where $\phi_{\mathrm{RIS-Rx}}$ represents the transmission angles on the RIS plane.
From (S3) and (S4), the total pathloss of the link via RIS is expressed by

$$P_{\mathrm{RIS}} = P_{\mathrm{Tx-RIS}} P_{\mathrm{RIS-Rx}} = \left(\frac{4\pi d_{\mathrm{Tx-RIS}} d_{\mathrm{RIS-Rx}}}{A\cos\phi_{\mathrm{Tx-RIS}}\cos\phi_{\mathrm{RIS-Rx}}}\right)^2. \qquad (S5)$$

In order to satisfy the condition that the total propagation loss of the path through the RIS is a level commensurate with the FSPL of the total propagation path length, the following

inequality must hold:

$$P_{\text{direct}} \geq P_{\text{RIS}}, \qquad s.t.\ \ d = d_{\text{Tx-RIS}} + d_{\text{RIS-Rx}}. \tag{S6}$$

By comparing (R1) and (R5) with $d = d_{\text{Tx-RIS}} + d_{\text{RIS-Rx}}$, we find that

$$\left(\frac{4\pi d}{\lambda}\right)^2 \geq \left(\frac{4\pi d_{\text{Tx-RIS}} d_{\text{RIS-Rx}}}{A \cos\phi_{\text{Tx-RIS}} \cos\phi_{\text{RIS-Rx}}}\right)^2$$

$$\frac{d^2}{\lambda^2} \geq \frac{{d_{\text{Tx-RIS}}}^2 {d_{\text{RIS-Rx}}}^2}{A^2 \cos^2\phi_{\text{Tx-RIS}} \cos^2\phi_{\text{RIS-Rx}}}$$

$$A \geq \frac{\lambda d_{\text{Tx-RIS}} d_{\text{RIS-Rx}}}{d_{\text{Tx-RIS}} + d_{\text{RIS-Rx}}} \cdot \frac{1}{\cos\phi_{\text{Tx-RIS}} \cos\phi_{\text{RIS-Rx}}} \geq \frac{\lambda d_{\text{Tx-RIS}} d_{\text{RIS-Rx}}}{d_{\text{Tx-RIS}} + d_{\text{RIS-Rx}}} \triangleq {r_{\text{Fresnel}}}^2, \tag{S7}$$

where $r_{Fresnel}$ is the Fresnel radius.

Assuming that the RIS is a square, the length of one side is given by $\sqrt{A}$. Therefore, from (R7), the required side length of the RIS is the radius of the 1st Fresnel zone, i.e., $\sqrt{A} \geq r_{\text{Fresnel}}$.

2. Quality factor of the antisymmetric mode

The impact of the LC thickness on the antisymmetric mode of bilayer cross-dipoles was examined in electromagnetic (EM) simulations. Figure S1a shows the LC thickness dependence of the antisymmetric mode peaks when the pattern is made of either a copper material with a finite conductivity of 5.8×10^{7} S m^{-1} or a perfect electrical conductor (PEC). Here, the loss tangent of the LC is set to be ideally 0. In the case of copper, a reduction in the LC thickness decreases the resonance peak amplitude. However, the peak remains when the PEC is used. The magnetic dipole (MD) excited in antisymmetric mode can be characterized by the magnetic surface impedance,

$$\boldsymbol{Z}_{\mathbf{s}} = \frac{2\eta(1 - T + R)}{1 + T - R}, \qquad (S8)$$

where R is the reflection coefficient, T is the transmission coefficient, and $\eta = \sqrt{\mu/\varepsilon}$ is the wave impedance of free space[2,3]. Figure S1b shows the real part of $\boldsymbol{Z}_{\mathbf{s}}$, derived from equation (S8), wherein the frequency is normalized by the peak frequency (f_{rMD}). It can be seen that an increase in the strength of the MD confinement in the LC layer results in a higher quality factor. Conversely, the quality factor of the MD for the copper pattern decreases as the LC layer becomes thinner, which is due to the ohmic loss of the copper collapsing the high-Q resonance peaks (Fig. S1c).

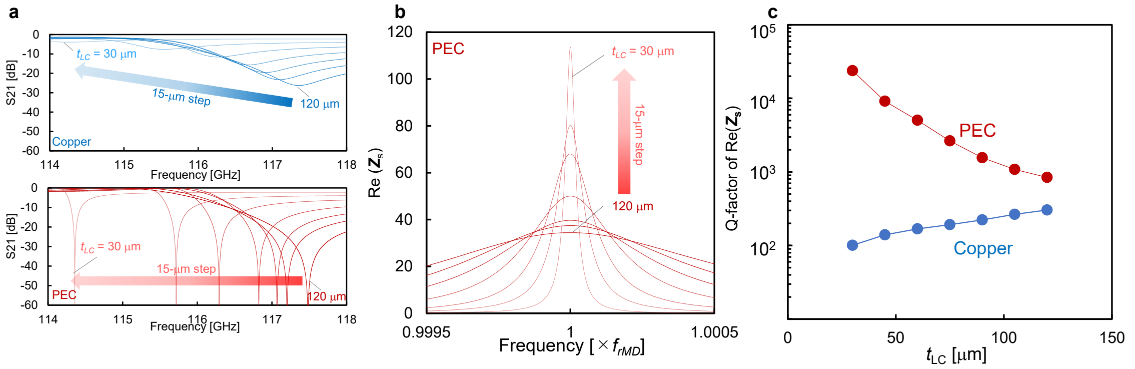

Fig. S1: Impact of liquid crystal (LC) thickness on the antisymmetric mode properties of a bilayer cross dipole

a Simulated transmittance for the antisymmetric mode of a bilayer cross dipole patterned with copper or PEC. The conductivity of the copper used in the simulations is 5.8×10^{7} S m-^{1}. **b** Dependence of the real part of Z_s for the perfect electrical conductor (PEC)-patterned bilayer cross dipole on LC thickness. **c** Dependence on LC thickness of the Q-factor of Z_s for the PEC- and copper-patterned bilayer cross dipole.

The effect of the loss tangent of a 30-μm-thick LC layer was also simulated. Figure S2 shows the simulation results for the change in Q factor when the metal conductivity or

loss tangent of the LC layer are varied. When the metal conductivity is 5.8×10^{7} S m^{-1}, Q factor of Re($\boldsymbol{Z}_s$) decreases to ~100. Similarly, when the loss tangent of the LC layer is 0.01, the Q factor of Re($\boldsymbol{Z}_s$) decreases to ~180. These results indicate that it is difficult to use the antisymmetric mode on an LC metasurface with an LC thickness of a few micrometers when it is made of a real material with finite conductivity and dielectric loss.

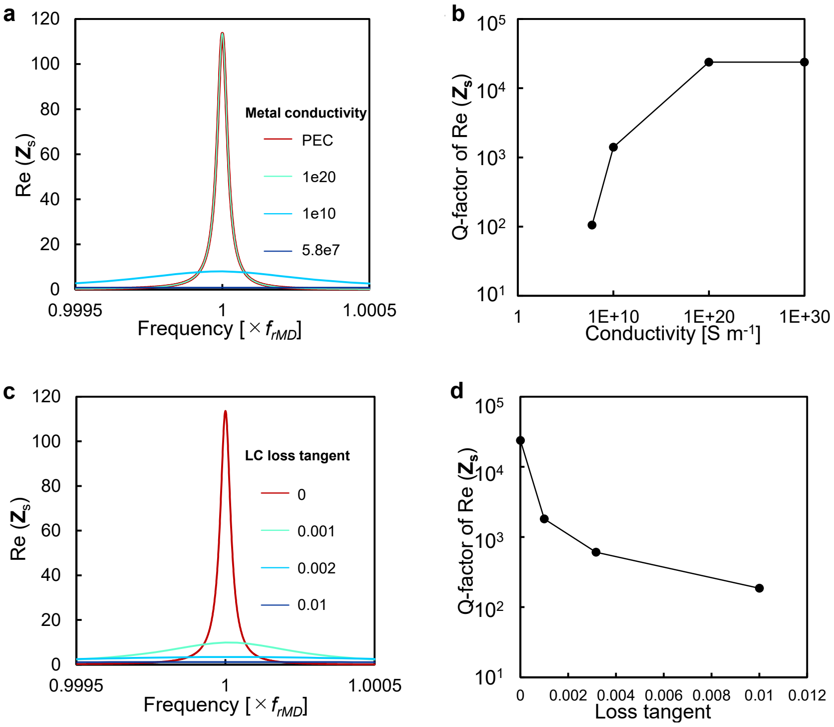

Fig. S2: Simulated Q factor of Re($\boldsymbol{Z}_s$) for bilayer cross dipole with 30-μm-thick liquid-crystal (LC) layer.

a Dependence of the real part of $\boldsymbol{Z}_s$ for a bilayer cross dipole with a lossless LC layer on metal conductivity. **b** Dependence on metal conductivity of Q-factor of $\boldsymbol{Z}_s$ for bilayer cross dipole with a lossless LC layer. **c** Dependence of the real part of $\boldsymbol{Z}_s$ for the perfect electrical conductor (PEC)-patterned bilayer cross dipole on the loss tangent of the LC layer. **d,** Dependence on the loss tangent of the LC layer of **the** Q-factor of $\boldsymbol{Z}_s$ for the PEC-patterned bilayer cross dipole.

3. Excited dipole in the circulating-current mode of the S-SRRs

In the circulating-current mode of S-SRR, from the cross-sectional view, no looped currents are excited that would produce MD (Fig. S3a). As shown in section 2, the electrical and magnetic resonance modes can be distinguished by characterizing the surface impedance in equation (S8) and surface admittance, Y_s, expressed by

$$\boldsymbol{Y}_s = \frac{2(1-T-R)}{\eta(1+T+R)}. \qquad (S9)$$

Figure S3b shows the real part of the simulated Y_s and Z_s derived from equations (S8) and (S9) for the circulating-current mode of S-SRR. The peak of Y_s corresponding to ED is around 115 GHz, where the circulating-current mode of the S-SRR is excited, while there is no Z_s peak corresponding to MD. This indicates that ED is excited in the S-SRR, unlike MD in the anti-symmetric mode of the bilayer cross dipole.

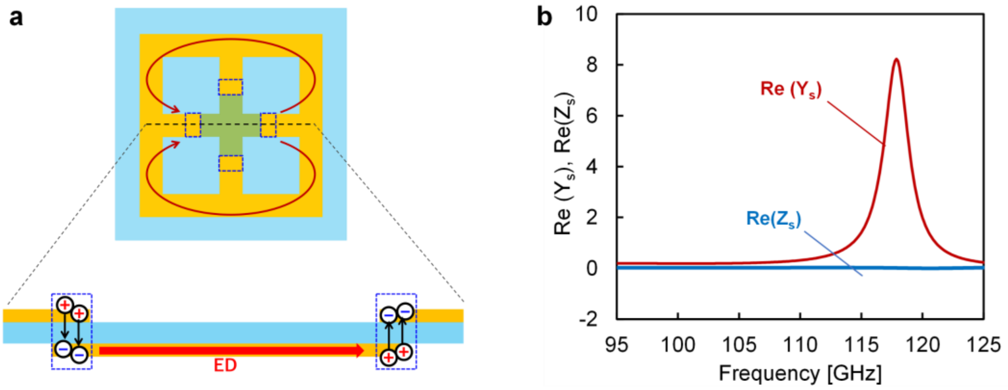

Fig. S3: Electric dipole excited in the circulating-current mode of the stepped split-ring resonator (S-SRR).

a Conceptual diagram of cross-sectional E field and **b** simulated surface admittance and impedance for circulating-current mode of S-SRR.

4. Effect of material properties on resonant characteristics

The effect of the metal conductivity, loss tangent of the glass substrate, and loss tangent of the LC layer on the resonant peaks was simulated to clarify the role of the material parameters in determining the modulation depth of the S-SRR (Fig. S4). For the ideal material parameters (PEC for metal, glass loss tangent of 0, LC loss tangent of 0), the resonance peak of the S-SRRs is ~80 dB. Each material parameter of the conductivity or loss tangent was varied, and it was found that the resonant peak becomes smaller as the metal conductivity decreases or as the loss tangents increases. For the materials mentioned in the main text (metal conductivity of 4.65×10^{7} S m^{-1}, glass loss tangent of 0.01, LC loss tangent of 0.014), the metal conductivity has the largest effect on the decrease in the resonant peak.

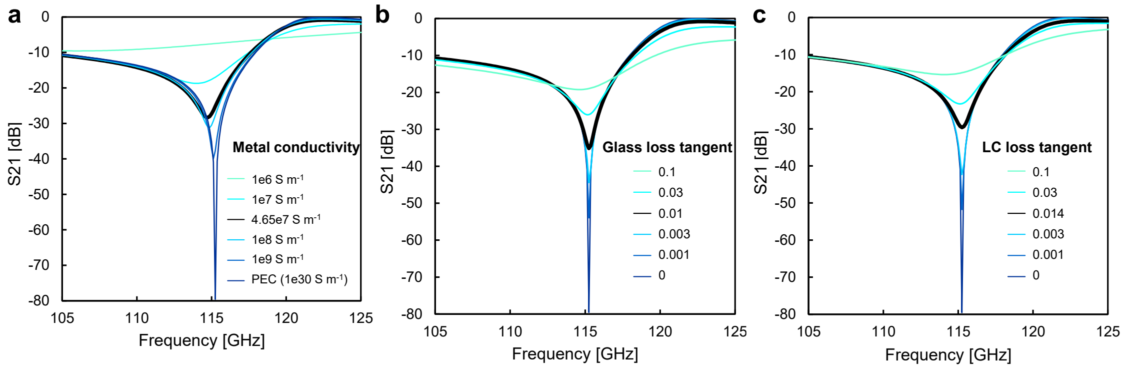

Fig. S4: Simulated transmittance for the stepped split-ring resonator (S-SRR) without the bias lines. **a** The metal conductivity, **b** loss tangent of glass substrate, or **c** loss tangent of liquid-crystal layer is varied with all the material parameters except for the changed ones being ideal.

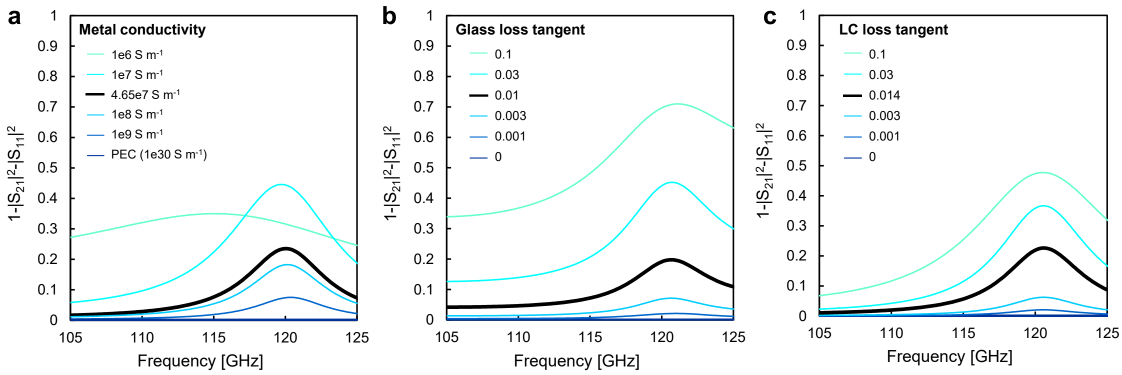

Fig. S5: Dissipated power in the stepped split-ring resonator (S-SRR) without the bias lines. **a** The metal conductivity, **b** loss tangent of glass substrate, or **c** loss tangent of liquid-crystal layer is varied with all the material parameters except for the changed ones being ideal.

Figure S5 shows the simulated power dissipated in the S-SRR ($1-|S_{21}|^2-|S_{11}|^2$) without the bias line, corresponding to the simulated transmittance in Fig. S5. The peaks of the dissipated power are at a higher frequency than the resonance peak of the transmittance (115 GHz), and for each material parameter used in the main text, the dissipated power at 115 GHz is less than 10% in all cases, while the power dissipated in the bias lines with 60 Ω/sq is 20% (Fig. 3f in the paper).

5. Channel model for metasurface-assisted path

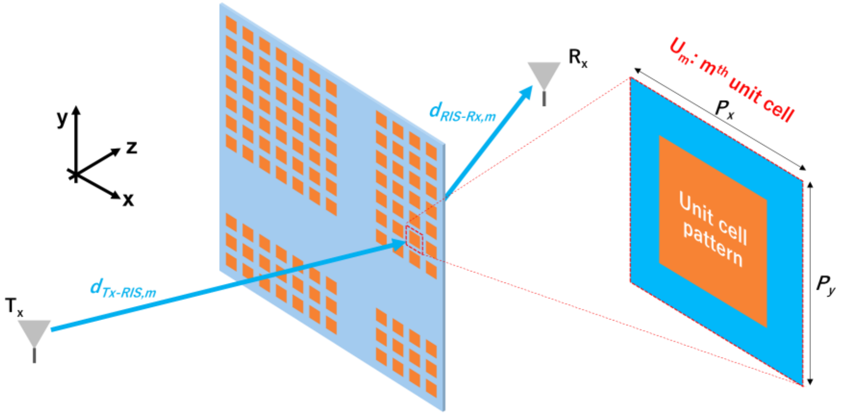

Fig. S6: Calculation model for the metasurface-assisted channel. This model is the basis for the calculated results in the main text.

To evaluate the validity of the experimental results, we used a numerical model of the path loss from the transceiver (Tx) through the metasurface to the receiver (Rx)[1]. Figure S6 shows the calculation model, where Tx is at $d_{\text{Tx-RIS},m}$ and Rx is at $d_{\text{RIS-Rx},m}$ from the mth unit cell of the metasurface. Let A_{U} denote the area of the unit cell. Each cell is assumed to be relatively small compared with the transmission distance, i.e., $A_{\text{U}} \ll d_{\text{Tx-RIS},m}, d_{\text{RIS-Rx},m}$. Accordingly, the effective areas of the mth unit cells on the Tx and Rx sides are defined by making a plane-wave approximation as follows:

$$A_{\text{Tx-RIS},m} = A_{\text{U}} \cos\phi_{\text{Tx-RIS},x,m} \cos\phi_{\text{Tx-RIS},y,m} , \quad \text{(S10)}$$

$$A_{\text{RIS-Rx},m} = A_{\text{U}} \cos\phi_{\text{RIS-Rx},x,m} \cos\phi_{\text{RIS-Rx},y,m} , \quad \text{(S11)}$$

where $\phi_{\text{Tx-RIS},x,m}$ and $\phi_{\text{Tx-RIS},y,m}$ represent the incident angles on the *xz* and *yz* planes, respectively, and $\phi_{\text{RIS-Rx},x,m}$ and $\phi_{\text{RIS-Rx},y,m}$ represent the transmission angles on the *xz* and *yz* planes, respectively.

Let $h_m \in \mathbb{C}$ denote the channel through the mth unit cell in the metasurface. From the Friis transmission formula, h_m is expressed as

$$h_m = \sqrt{\frac{A_{\text{RIS-Rx},m}}{4\pi d_{\text{RIS-Rx},m}^2}} e^{-\frac{j2\pi}{\lambda} d_{\text{RIS-Rx},m}} \times b_m \times \sqrt{\frac{A_{\text{Tx-RIS},m}}{4\pi d_{\text{Tx-RIS},m}^2}} e^{-\frac{j2\pi}{\lambda} d_{\text{Tx-RIS},m}} \quad \text{(S12)}$$

$$= \frac{\sqrt{A_{\mathrm{Tx-RIS},m} A_{\mathrm{RIS-Rx},m}}}{4\pi d_{\mathrm{Tx-RIS},m} d_{\mathrm{RIS-Rx},m}} b_m e^{-\frac{j2\pi}{\lambda}(d_{\mathrm{Tx-RIS},m}+d_{\mathrm{RIS-Rx},m})}, \tag{S13}$$

where $b_m \in \{0, 1\}$ denotes the binary intensity profile corresponding to the mth unit cell.

As a result, the path loss of the channel through the metasurface is expressed as

$$P_{\mathrm{loss}} = \left| \sum_{m=1}^{M} h_m \right|^2 . \tag{S14}$$

Note that this derivation does not account for the polarization or power loss of transmitting through the metasurface.

6. Far-field validation with reduced aperture

To confirm the operation of the fabricated LC metasurface and validate the channel-model-based calculations under far-field conditions, an additional experiment was conducted using a reduced aperture configuration. The effective aperture of the LC metasurface was limited to 30 mm × 30 mm by surrounding absorbers (Fig. S7). The far-field condition can be expressed as:

$$R_{\mathrm{far}} > \frac{2D^2}{\lambda}, \qquad (S15)$$

where D is the diameter of the aperture and λ is the wavelength. For D = 30 mm and λ = 2.6 mm (115 GHz), the far-field distance is approximately 0.69 m. To ensure sufficient margin, the measurement plane was placed at 1 m from the metasurface plane. Figure S8a compares the measured electric-field distribution in the xy-plane at z = 1 m for the all-on state and the collimating control state with the calculation. The measured results show good agreement with the calculated distribution. Figure S8b shows the electric-field profiles along y = 0 mm, indicating that the gain improvement due to collimation is approximately 6 dB—limited by the reduced aperture size—but the measured and calculated gains match closely. These results validate the operation of the fabricated LC metasurface and the channel-model-based calculations under far-field conditions.

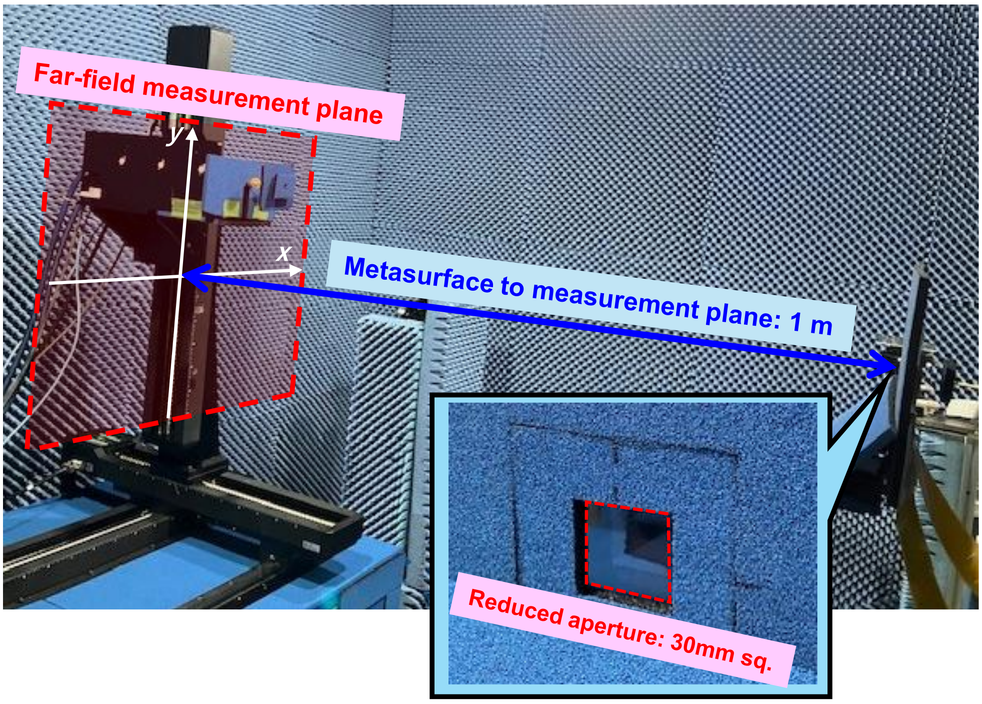

Fig. S7: Experimental setup for far-field validation using a reduced aperture (30 mm × 30 mm).

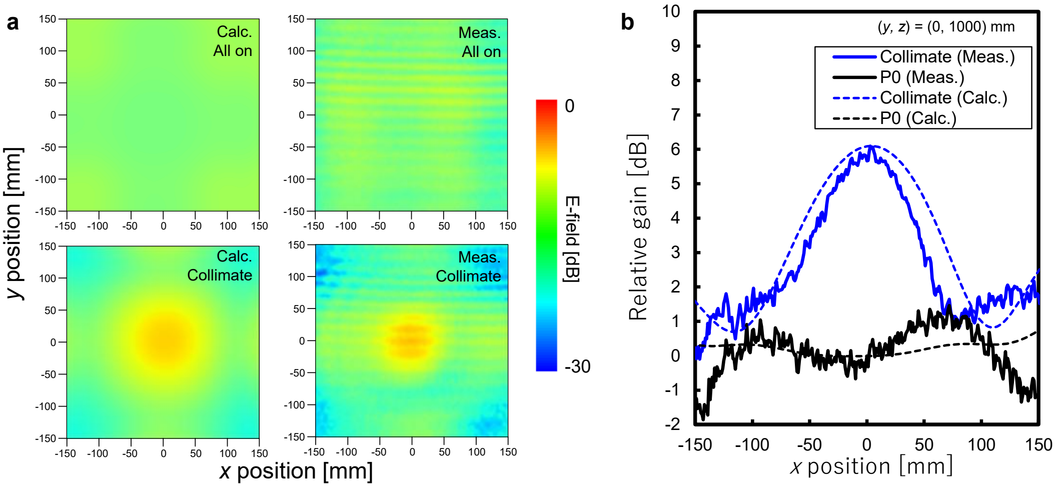

Fig. S8: Comparison of measured and simulated electric-field profiles at $z = 1$ m for the reduced aperture.

a Measured and calculated electric-field distribution on the xy plane at z = 1 m for all-on and collimating control. **b** Measured and calculated relative gain along $y = 0$ mm, showing ~6 dB gain due to collimation and good agreement between measurement and calculation.

7. Polarization and incident angle dependency of the S-SRRs

To interpolate the results from the two orthogonal linear polarizations, we performed full-wave simulations and measurements by changing the polarization of the incident wave from 0 to 90 degrees in 15-degree steps (Fig. S9). As described in the main text, the difference in the ITO length resulted in variations in the resonance peak intensity at 0 degrees (*x*-polarization) and 90 degrees (*y*-polarization). The additional simulations and measurements serve to interpolate between these values.

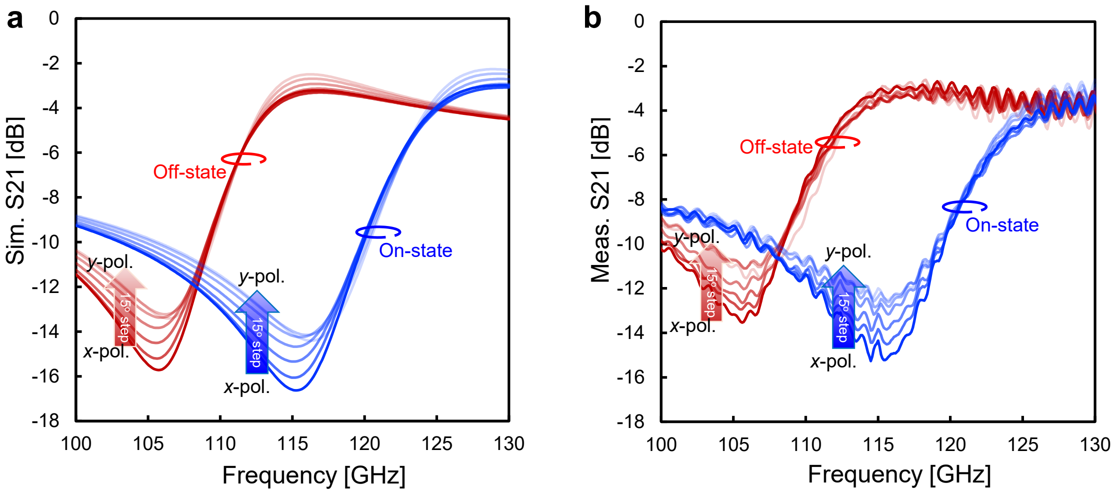

Fig. S9: Polarization dependency of the transmittance for the stepped split-ring resonators (S-SRRs). **a** Simulated and **b** measured transmittances, in which the polarization of the incident waves is varied from *x*-polarization to *y*-polarization in 15-degree steps.

Furthermore, to assess performance under practical conditions, full-wave simulations were conducted for oblique incidence angles ranging from 0° to 60° for both TE- and TM-polarized waves (Fig. S10). For TE-polarized incidence, increasing the angle resulted in higher insertion loss in both on-state and off-state, accompanied by a deeper resonance peak. In contrast, for TM-polarized incidence, increasing the angle led to lower insertion loss and a shallower resonance peak. The modulation depth at 115 GHz exhibited opposite trends for TE and TM polarization; however, even at 60°, the decrease for TM polarization was limited to approximately 2 dB. These results indicate that the proposed structure maintains effective performance over a wide range of incident angles.

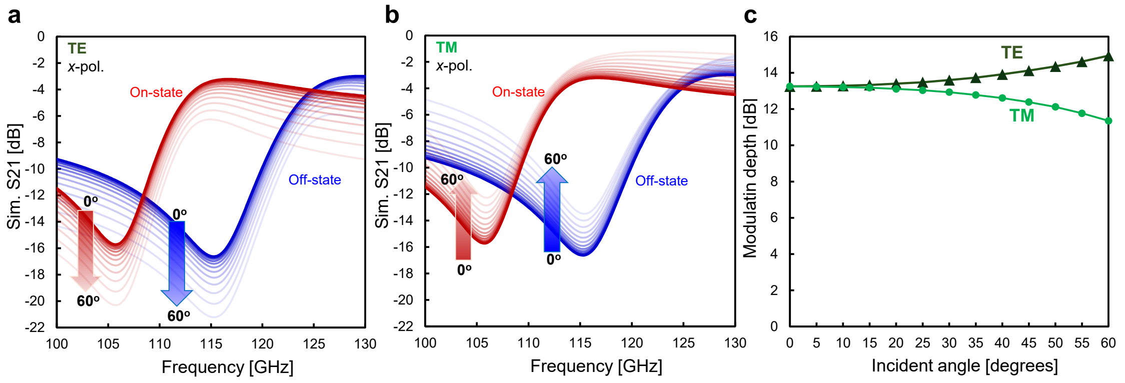

Fig. S10: Incident angle dependency of the transmittance for the stepped split-ring resonators (S-SRRs).

a Simulated transmission spectra for TE-polarized incidence and **b** TM-polarized incidence at angles from 0° to 60°. **c** Modulation depth on the incidence angle for TE- and TM-polarized incidence at 115 GHz.

8. Line-by-line matrix control method

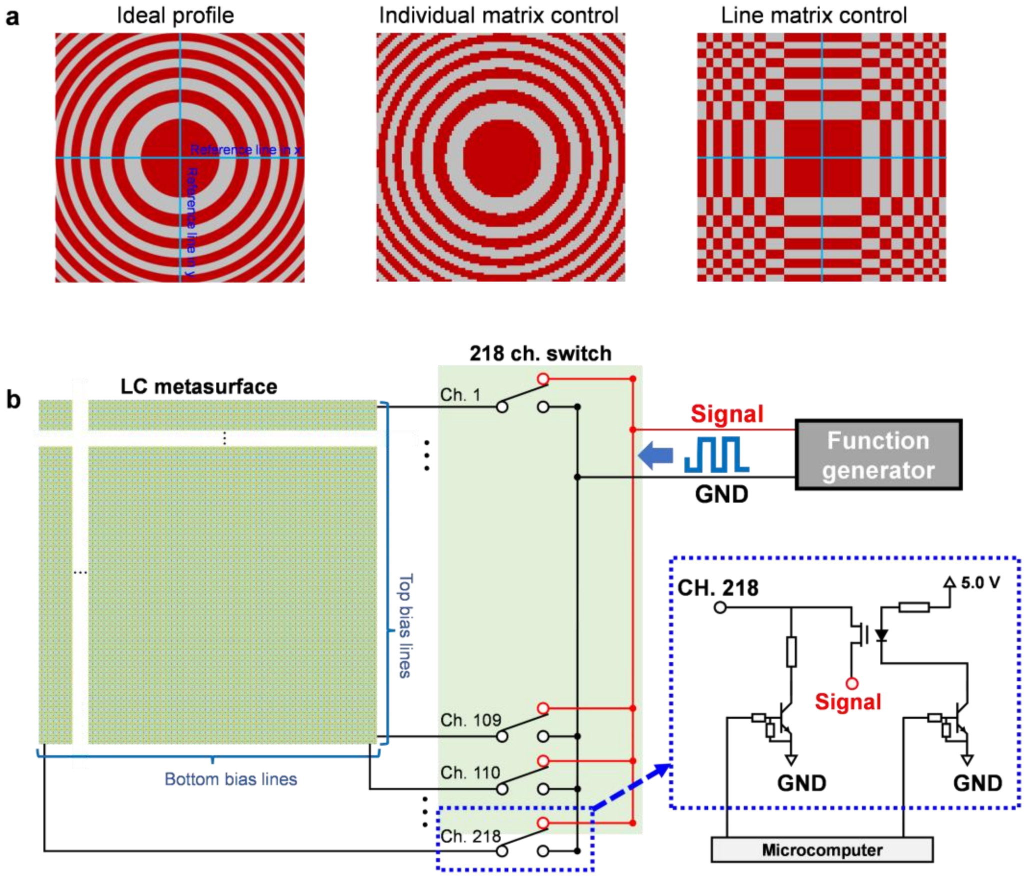

Fig. S11: Configuration of the line matrix control.

a Control patterns of individual matrix control, line matrix control, and their original ideal matrix for collimating the incident waves. **b** Schematic connections between fabricated liquid-crystal metasurface and the 218-channel switch used in this study.

The intensity profiles for P0 to P9 were designed by taking account of differences in the path length between the phase centers of port 1 and the position at which the transmission waves were to be directed through each cell of the LC metasurface. Figure S11a shows the P1 control pattern, its intrinsic ideal profile, and the individual matrix control profile. The line matrix control applies signals to each row and column line rather than controlling each cell individually. The binary profile of control channels for each row and column is determined on the basis of the reference position exhibiting the least phase change in the continuous wavefront obtained from the differences in the path length. According to the binary state on the reference lines in the *x*- and *y*-directions passing through the reference position, a signal is applied to each line using the 218-channel switch and function

generator, as illustrated in Fig. S11b. The switch for connecting to the signal or ground in each channel is composed of two MOSFET (metal-oxide-semiconductor field-effect transistor)-based semiconductor relays. The top and bottom bias lines of the LC metasurface, each with 109 channels, are switched to be connected to the signal or the ground of the function generator. Subsequently, the 1-kHz square wave generates a two-dimensional intensity profile like the P1 control pattern. It should be noted that the modulation profile employed in this study is not guaranteed to be the optimal discrete control. When quantizing wavefronts as in the binary modulation used in this study, in which the control pattern combinations number is 2^{218} for the 218 binary controlled channels, finding the optimized modulation profile is a discrete optimization problem. This makes the optimization of the modulation profile for each angle an NP-hard problem[4]. To further improve the RIS performance, it is important to investigate discrete optimization methods that would result in an optimized wavefront in the RIS plane.

9. Estimated diffraction efficiency for RIS with all-on configuration

The amplitude modulation profile changes depending on the relative positions of the Tx, RIS, and Rx. In most cases, it is necessary to change the direction and focus of the transmitted wave by using amplitude modulation in which on-state and off-state cells coexist in approximately equal proportion, and their theoretical diffraction efficiency is 10%. However, under certain conditions, such as when the Tx, RIS, and Rx are aligned on a straight line, the optimal amplitude modulation profile may be all-on rather than partially-on. When the Tx and RIS (and the RIS and RX) are separated by a sufficient distance, wherein the difference in path lengths between the transmitting point and the receiving point through each cell of the RIS is less than π, in other words, when the RIS size is smaller than the 1st Fresnel zone, the optimal amplitude modulation is all-on. An aperture with a difference in path lengths through each cell of less than π is equivalent to or larger than a 1-bit phase (0/π) modulated aperture whose phase quantization error is less than π. Therefore, the diffraction efficiency when all-on is the optimal amplitude modulation profile is 41% or more. Furthermore, when the difference in path lengths between the Tx and Rx via each cell is less than π/2, the diffraction efficiency of all-on is greater than that for 2-bit phase modulation, i.e., 81% or more, and the diffraction efficiency of all-on gradually approaches 100% as the distance increases.

10. Effect of unit cell size on the steering characteristics

When a discrete wavefront is formed, such as in a metasurface, the accuracy of the beam control is correlated with the wavefront resolution, i.e., the cell size. Figure S12 shows the calculated deflection angle error of the transmitted wave through the intensity profile of the metasurface with respect to the designed steering angle when the cell size is changed. The designed angle is varied from 0 to 5 degrees and from 25 to 30 degrees in 1-degree steps, and the size of the metasurface is constant at 70 mm square regardless of the cell size. As the cell size increases, the wavefront resolution degrades, and the error in the deflection angle relative to the design angle increases. The error also tends to increase as the designed deflection angle increases. For larger angles, the spatial frequency of the amplitude modulation is increased, and the error between the ideal profile and the one formed by RIS with the finite control unit size becomes large.

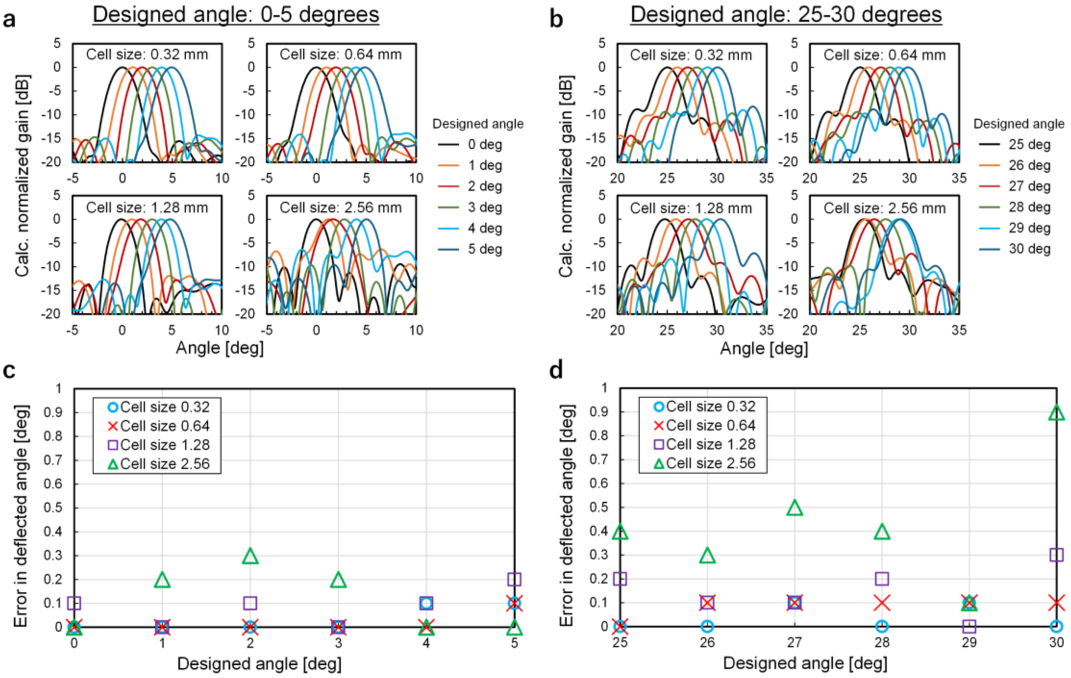

Fig. S12: Calculated deflection angle of the transmitted waves through the intensity profile of the metasurface with respect to the designed steering angle and the cell size.

a Calculated far-field beam patterns for designed angle from 0 to 5 degrees and **b** 25 to 30 degrees with respect to the cell size. **c** Error in deflection angle relative to designed angle from 0 to 5 degrees and **d** 25 to 30 degrees with respect to cell size.

For instance, in the binary pattern for a 30-degree deflection of the one-dimensionally collimated waves, the binary pattern with a resolution of 0.64 mm has a total error width

of 8.2 mm compared with the ideal binary profile, which is 1.65 times wider than that for 0-degree deflection. (Fig. S13). Although the unit cell of the fabricated LC metasurface is 320-μm square, its resolution of the intensity profile is 640 μm since two rows or columns are bundled together into one channel. Steering performance could be improved by increasing the number of channels to enable control of each row and column or by reducing the unit cell size.

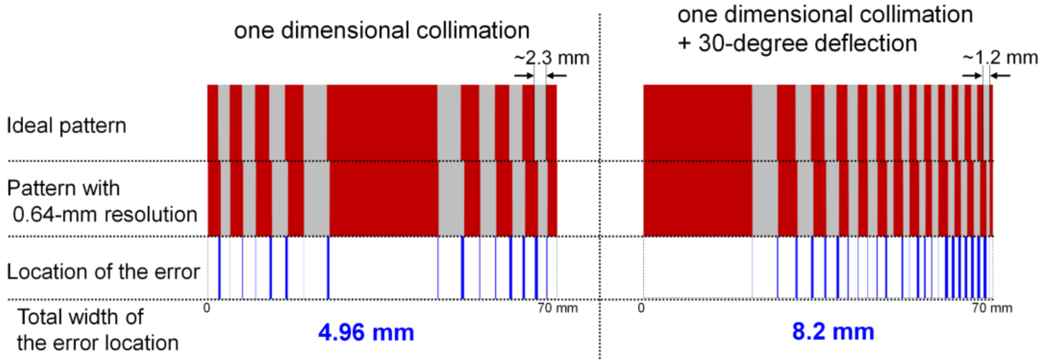

Fig. S13: Diagram showing degree of discrepancy between the formed and ideal binary profile depending on the cell size and steering angle.

11. Near-field distribution in magnetic-field plane

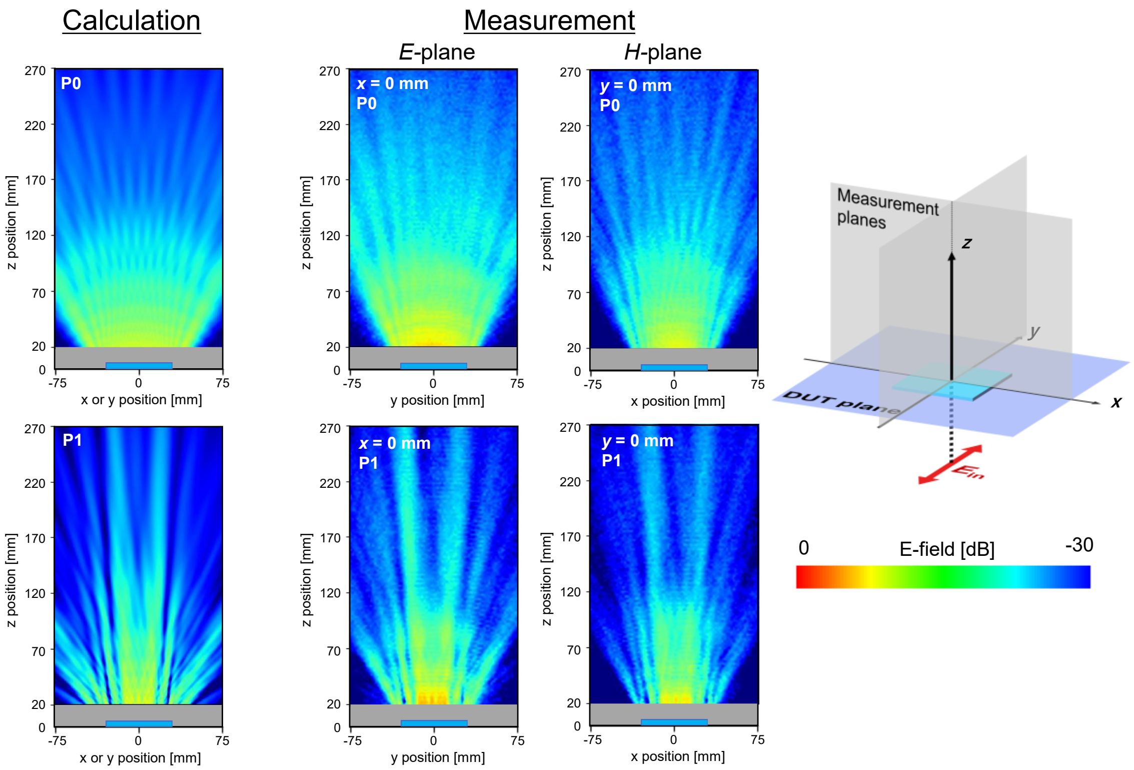

Fig. S14: Calculated and measured *E*-field distributions for control patterns P0 and P1.

Figure S14 shows the calculated and measured near-field distributions for control patterns P0 and P1 in both the *xz* and *yz* planes for *y*-polarized incident waves. In the *yz* plane corresponding to the electric-field plane and the *xz* plane corresponding to the magnetic-field plane, the widely spread transmitted waves for P0 were collimated by the P1 control pattern. The measured results agreed well with the calculations.

12. Measurement of response time of LC orientation change

The multi-channel switch used in the feasibility verification described in the main text is not appropriate for the evaluation of the response time. The rise and fall times of the LC metasurface are determined by the response time of the LC when the control driver is constructed like an LCD driver. Therefore, we measured the response time of the LC orientation change through the change in transmittance in the lightwave band. Figure S15 shows the measured transmittance of light, which varies with the LC orientation, for a sample with an LC layer thickness of 3.5 μm, as is the case for the proposed structure. In this experiment, 1-kHz square-wave signal with a peak voltage (V_p) of 8 V was applied, and the temperature was varied from 25°C to 60°C. During the rise time when V_p changed from 0 V to 8 V, the response time became shorter as the temperature increased; it was approximately 3 ms at room temperature (25°C) and approximately 1 ms at 60°C. Similarly, during the fall time when V_p changed from 8 V to 0 V, the response time became shorter as the temperature increased; it was approximately 50 ms at 25°C and approximately 30 ms at 60°C.

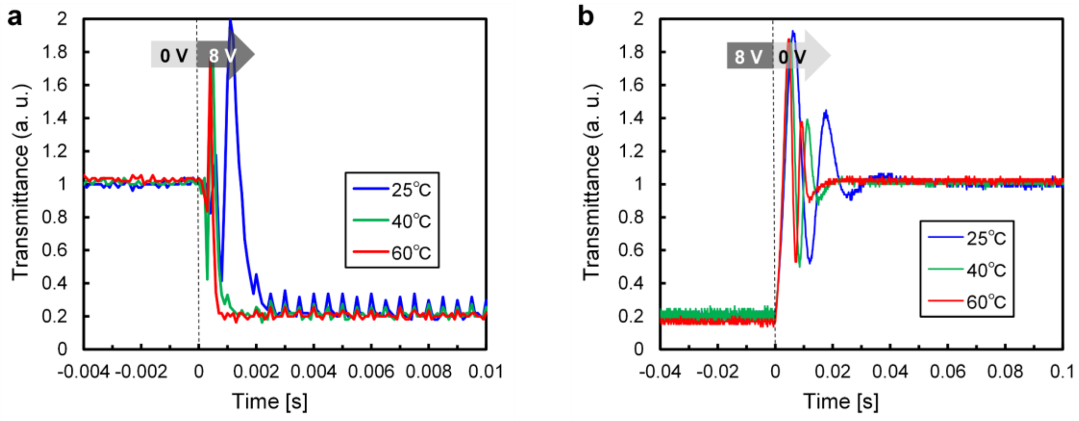

Fig. S15: Measured transmittance of light for determining the response time of the liquid-crystal (LC) orientation change. a Rise time measured by varying V_p from 0 V to 8 V. **b** Fall time measured by varying V_p from 8 V to 0 V. The LC thickness of the sample is 3.5 μm, which is the same as that of the LC metasurface described in the main text.

To further analyze the dynamic behavior under the proposed metasurface configuration, we simulated the LC director dynamics using LCD Master (Shintech Optics) based on S-SRR's geometry and material properties of DHB-012 (DIC Corp., Japan). The simulation results, shown in Fig. S16, indicate that when V_p changes from 0 V to 8 V, the director tilt

reaches approximately 90° within 3 ms and stabilizes thereafter. When V_p returns from 8 V to 0 V, the tilt decreases below 30° within 50 ms and approaches near-horizontal alignment (<10°) by 100 ms. These results are consistent with the measured optical response and highlight the advantage of using an LCD-grade LC thickness for achieving millisecond-scale switching.

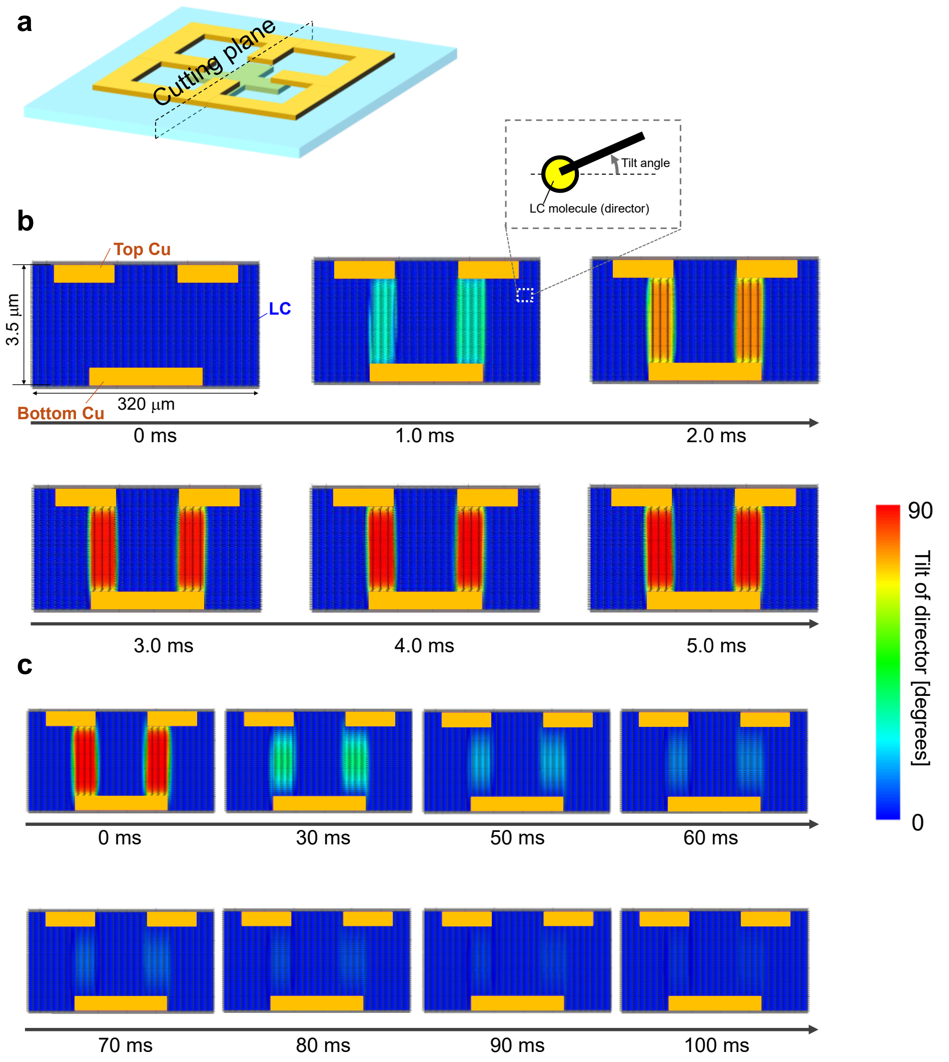

Fig. S16: Simulation results of liquid-crystal (LC) molecule dynamics at 25°C in the stepped split-ring resonator (S-SRR).

a S-SRR model used in the simulation of LC director dynamics. **b** Director tilt distribution in the cross-section of the LC layer of the S-SRR for the rise time and **c** the fall time evaluation.

13. Detailed geometric parameters of the unit cell

The detailed geometrical parameters of the unit cell structures used in the EM simulations and experiments are illustrated in Fig. S17 and Table S1. In the simulations, the chromium layer was treated as copper to reduce the computational load.

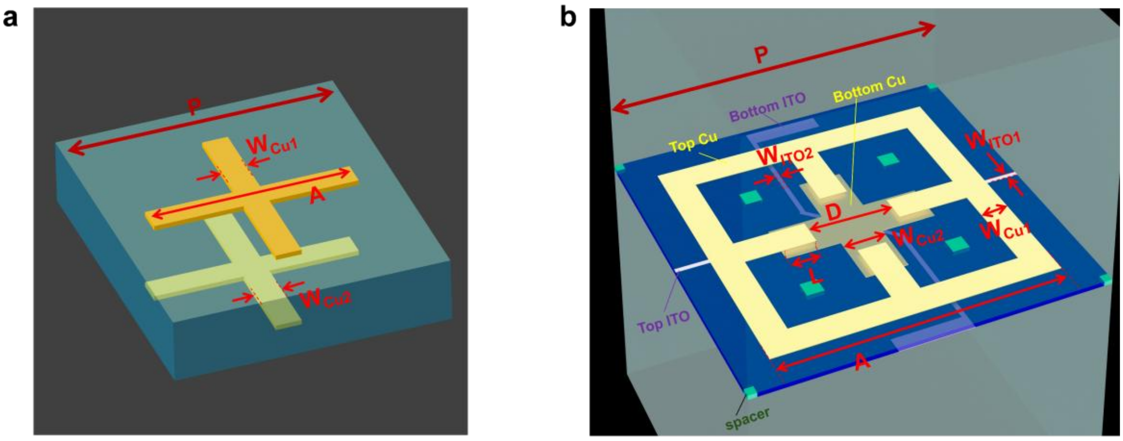

Fig. S17: Geometry and dimensions of the unit cell used in this study.

a Bilayer cross dipole cell. **b** stepped split-ring resonator (S-SRR) cell.

Table S1 Geometric parameters of the cells used in the simulations and measurements.

		P	A	W_{Cu1}	W_{Cu2}	D	L	W_{ITO1}	W_{ITO2}
Fig. 2a-c	Sim.	950 μm	770 μm	50 μm	50 μm				
Fig. 2g	Sim.	3.5 mm	2.7 mm	150 μm	150 μm	1 mm	60 μm		
Fig. 2h	Sim.	1.2 mm	1 mm	80 μm	100 μm	400 μm	40 μm		
Fig. 2i	Sim.	320 μm	260 μm	30 μm	45 μm	90 μm	35 μm		
Fig. 4d	Sim.	320 μm	260 μm	30 μm	45 μm	90 μm	32 μm	5 μm	5 μm
Fig. 4d	Meas.	320 μm	260 μm	30 μm	45 μm	90 μm	32 μm	5 μm	5 μm

14. LC material information

Table S2 lists the permittivity and loss tangent data at 10 GHz and λ of 589 nm for the nematic LC material used in this study as disclosed by the supplier (DHB-012, DIC Corp.). The changes in $\varepsilon_{/\!/}$, $\varepsilon_{\perp}$, and tunability from 10 GHz to the lightwave band are approximately 2%, 10%, and 7%, respectively, suggesting that nearly constant dielectric properties can be obtained within a range of several percent of the relative bandwidth typically necessary for mobile communication systems.

Table S2 Permittivity and loss tangent data measured at 10 GHz and λ of 589 nm.

	DHB-012 (DIC corp.)	
	10 GHz	589 nm
$\varepsilon_{/\!/}$	3.84	3.76
$\varepsilon_{\perp}$	2.65	2.35
Tunability $(\varepsilon_{/\!/} - \varepsilon_{\perp})/\varepsilon_{/\!/}$	30.9%	37.6%
$\tan\delta_{/\!/}$	0.0063	-
$\tan\delta_{\perp}$	0.0144	-
Transition temperature	160	

15. Effect of alignment error on the S-SRRs' characteristics

The alignment accuracy between the top and bottom metal is one of the important manufacturing parameters to ensure the reproducibility of the device. Figure S18 shows photos of two fabricated samples and their transmission characteristics, in which sample A, which is discussed in the main text, exhibits a small alignment error of ~4 μm and sample B exhibits a large alignment error of ~15 μm in the *y* direction. When an error occurs in the alignment, the overlap area between the top and bottom metal decreases, reducing the capacitance component of the S-SRR. This causes the resonance frequency to shift to a higher frequency than the designed one, consequently resulting in poor manufacturing yields. LCD manufacturers have achieved high-precision alignment (error of 1 μm or less) with repeatability. The proposed structure, which can be fabricated using the same process as LCDs, is thus considered to exhibit sufficient reproducibility in the mass-production process.

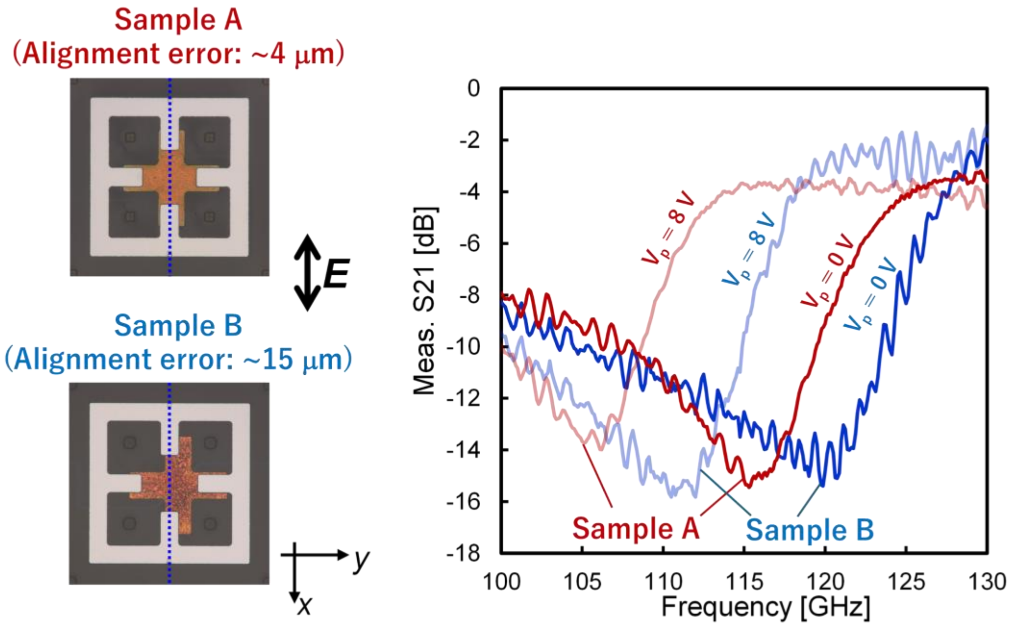

Fig. S18: Photos of two fabricated samples and their transmission characteristics. Sample A with a small alignment error of ~4 μm is discussed in the main text. Sample B exhibits a large alignment error of ~15 μm in the *y* direction.

16. Measurement uncertainty and positioning sensitivity

To ensure the reliability and reproducibility of the reported measurements, additional evaluations were conducted focusing on uncertainty and probe positioning accuracy. All measurements in this paper were performed in an anechoic chamber to maintain a controlled propagation environment. Repeated measurements were carried out for the collimating pattern of Supplemental Section 6. Figure S19 shows the repeated measurement results and the variation in received power across them. The measured uncertainty including unintended reflections was confirmed to remain below ±0.2 dB, indicating high measurement stability.

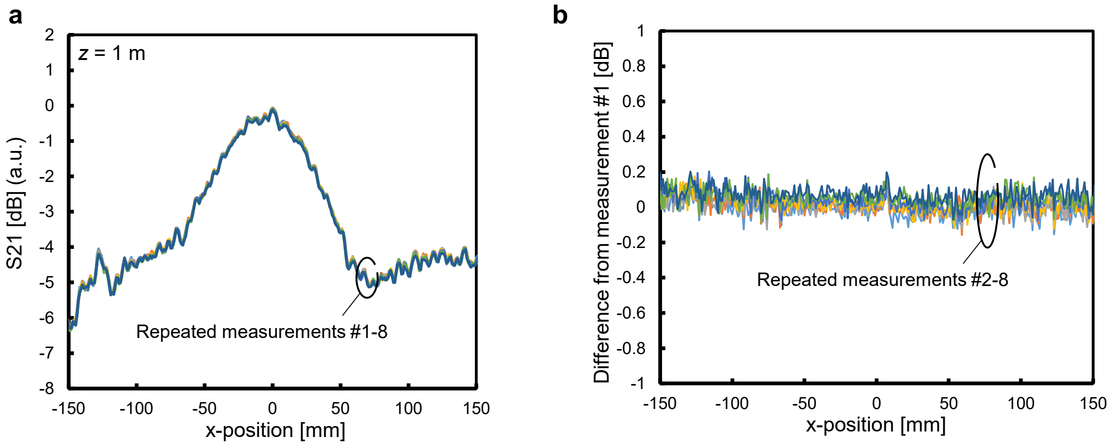

Fig. S19: Measurement uncertainty evaluation.

a Repeated far-field measurement results for the collimating pattern with reduced aperture at 115 GHz.

b Variation in received power across repeated scans, confirming measurement uncertainty remains below ±0.2 dB.

The sensitivity of probe positioning was examined under a focusing configuration, which represents a highly sensitive condition to the probe position. Figure S20 shows the dependency of received power at the focal point on the displacement of the Tx probe antenna in both horizontal (parallel to the metasurface plane) and vertical (normal to the metasurface plane) directions. The designed positions of the Tx antenna and focal length were 100 mm and 50 mm from the LC metasurface, respectively. The results indicate that sensitivity in the horizontal direction is higher than in the vertical direction. Nevertheless, even in the horizontal direction, the variation in received power remains within approximately 1 dB when the probe position is maintained within ±2 mm of the design position. Considering that the used positioning system (SGSP46, SIGMAKOKI Co., Ltd.)

provides an accuracy of 6 μm, the reproducibility of probe positioning is sufficiently ensured.

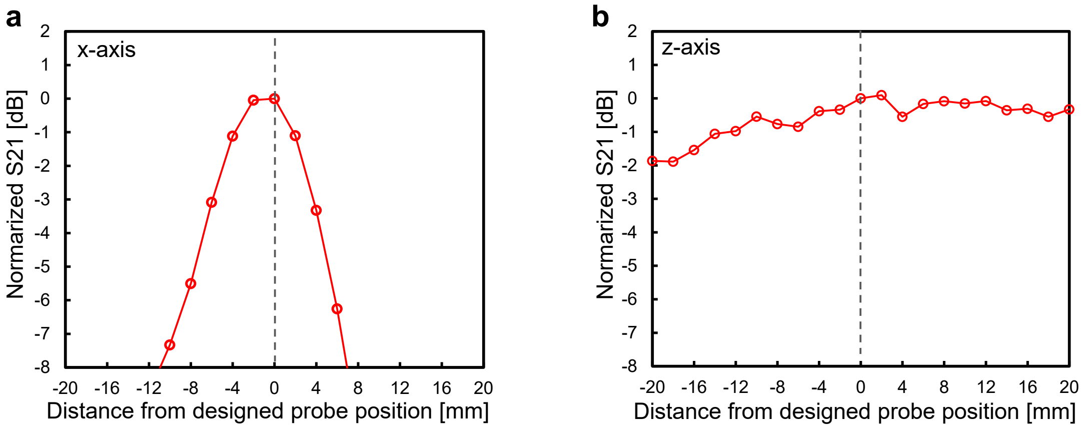

Fig. S20: Positioning sensitivity evaluation.

Received power variation at the focal point on the probe displacement **a** in the horizontal direction (parallel to the metasurface plane) and **b** in the vertical direction (normal to the metasurface plane).

References

1. Tang, W. *et al.* Wireless Communications with Reconfigurable Intelligent Surface: Path Loss Modeling and Experimental Measurement. *IEEE Trans. Wirel. Commun.* **20**, 421–439 (2021).
2. Holloway, C. L., Mohamed, M. A., Kuester, E. F. & Dienstfrey, A. Reflection and transmission properties of a metafilm: With an application to a controllable surface composed of resonant particles. *IEEE Trans. Electromagn. Compat.* **47**, 853–865 (2005).
3. Pfeiffer, C. & Grbic, A. Metamaterial Huygens' surfaces: Tailoring wave fronts with reflectionless sheets. *Phys. Rev. Lett.* **110**, 197401 (2013).
4. Shtaiwi, E. *et al.* Sum-Rate Maximization for RIS-Assisted Integrated Sensing and Communication Systems With Manifold Optimization. *IEEE Trans. Commun.* **71**, 4909–4923 (2023).
